# Plasminogen Activator Inhibitor-1 (PAI-1) deficiency predisposes to depression and resistance to treatments

**DOI:** 10.1186/s40478-019-0807-2

**Published:** 2019-10-14

**Authors:** Hélène Party, Cléo Dujarrier, Marie Hébert, Sophie Lenoir, Sara Martinez de Lizarrondo, Raphaël Delépée, Claudine Fauchon, Marie-Christine Bouton, Pauline Obiang, Olivier Godefroy, Etienne Save, Laurent Lecardeur, Joëlle Chabry, Denis Vivien, Véronique Agin

**Affiliations:** 1 UNICAEN, INSERM, INSERM UMR-S 1237, Physiopathology and Imaging of Neurological Disorders, Normandie University, 14000 Caen, France; 2 University of Trento, Center for Mind/Brain Science, 38068 Rovereto, Italy; 3 UNICAEN, PRISMM core facility, SF4206 ICORE, Comprehensive Cancer Center F. Baclesse, Normandie University, 14000 Caen, France; 4 UNICAEN, Centre Universitaire de Ressources Biologiques (CURB), Normandie University, 14000 Caen, France; 5 INSERM UMR-S U1148, Laboratory for Vascular and Translational Science, Paris Diderot University, Sorbonne Paris Cité University, 75013 Paris, France; 6 Department of Neurology and Laboratory of Functional Neurosciences, EA 4559, Amiens University Hospital, 80054 Amiens, France; 7 CNRS, LNC, Laboratory of Cognitive Neuroscience UMR 7291, Aix Marseille University, 13331 Marseille, France; 8 UNICAEN, EA 7466, Imagery and Therapeutic Strategy in Schizophrenia, Normandie University, 14000 Caen, France; 9 University Hospital, Adult Psychiatric Unit, Mobile Intensive Care Unit, 14000 Caen, France; 10 Institut de Pharmacologie Moléculaire et Cellulaire, UMR 7275 CNRS-Université Côte d’Azur, Sophia Antipolis, 06560 Valbonne, France; 11 Caen Normandie Hospital, Department for Clinical Research, 14000 Caen, France

**Keywords:** Major depressive disorder, Plasminogen activator Inhibitor-1, Serotonin, Dopamine, Escitalopram, Fluoxetine, Genetic model of depression, Resistance to selective serotonin reuptake inhibitors

## Abstract

Major depressive disorder (MDD) is one of the most frequent psychiatric illnesses, leading to reduced quality of life, ability to work and sociability, thus ranking among the major causes of disability and morbidity worldwide. To date, genetic and environmental determinants of MDD remain mostly unknown. Here, we investigated whether and how the Plasminogen Activator Inhibitor-1 (PAI-1) may contribute to MDD. We first examined the phenotype of PAI-1 knockout (PAI-1−/−) and wild-type (PAI-1+/+) male mice with a range of behavioral tests assessing depressive-like behaviors (*n* = 276). We next investigated the mechanisms relating PAI-1 to MDD using molecular, biochemical and pharmacological analyzes. We demonstrate here that PAI-1 plays a key role in depression by a mechanism independent of the tissue-type Plasminogen Activator (tPA) – Brain-Derived Neurotrophic Factor (BDNF) axis, but associated with impaired metabolisms of serotonin and dopamine. Our data also reveal that PAI-1 interferes with therapeutic responses to selective serotonin reuptake inhibitors (escitalopram, fluoxetine). We thus highlight a new genetic preclinical model of depression, with the lack of PAI-1 as a factor of predisposition to MDD. Altogether, these original data reveal that PAI-1 should be now considered as a key player of MDD and as a potential target for the development of new drugs to cure depressive patients resistant to current treatments.

## Introduction

Major depressive disorder (MDD) is one of the most frequent psychiatric illnesses, leading to reduced quality of life, ability to work and sociability, thus ranking among the major causes of disability and morbidity worldwide [19, 40, 50]. To date, genetic and environmental determinants of MDD remain mostly unknown [8, 15, 27, 34, 41]. MDD is defined by major depressive episodes (MDE) which are characterized by five or more out of the nine following symptoms [2, 49]: depressed mood*, markedly diminished interest or pleasure* (anhedonia), change in weight or appetite, insomnia or hypersomnia, psychomotor retardation or agitation, fatigue or loss of energy, feelings of worthlessness or excessive or inappropriate guilt, impaired concentration or indecisiveness, thoughts of death or suicidal ideation or attempt (* one or two of these symptoms must be present). These symptoms must induce altered functioning, cause significant distress or impairment, and should not be attributed to a drug substance or medical condition. MDD is diagnosed only if MDE is not better explained by a psychotic disorder and if there is no history of mania or hypomania.

Current pharmacological treatments mainly consisting in selective serotonin reuptake inhibitors (SSRIs) are effective, however approximately 30–40% of patients are resistant to such treatments [1, 13, 16, 41, 51, 52]. Moreover, even in responsive people, effects of antidepressants are often delayed and induce disabling side effects [23, 31]. Therefore, there is a need for a better understanding of the pathophysiology of MDD in order to propose innovative treatments.

An abundant literature suggests that the Plasminogen Activator Inhibitor-1 (PAI-1) could be involved in MDD. For example, PAI-1 is found in brain structures related to mood regulation (e.g. amygdala, hippocampus, prefrontal cortex) and in blood circulation [6, 22, 30, 36, 45]. Several clinical studies have reported increased levels of plasmatic PAI-1 in depressed patients [14, 22, 24, 32]. Convergent with these findings, Tsai et al. [48] demonstrated that the genetic variants of *SERPINE1* gene (coding PAI-1) in humans may increase MDD susceptibility and decrease the acute therapeutic response to SSRIs. At the preclinical scale, increased concentrations of cerebral PAI-1 have been found in rats exposed to a chronic unpredicted mild stress (CUMS) procedure [45]. All together, these studies suggest that PAI-1 could be a biomarker of depression, although its direct and precise implication in MDD pathogenesis has not been demonstrated.

The possible implication of PAI-1 in the pathogenesis of depression also emerged from the neurotrophic hypothesis of depression [3, 18, 37]. Among neurotrophins, Brain-Derived Neurotrophic Factor (BDNF) has been repeatedly shown to be implicated in MDD as well as in the mechanisms of action of antidepressants [4, 9, 37, 47, 54]. BDNF arises from a precursor, proBDNF, which is proteolytically cleaved by the tissue-type Plasminogen Activator (tPA) – plasminogen system to produce the mature protein, mBDNF [26, 35]. Whereas mBDNF facilitates long-term potentiation and prevents apoptosis, the proBDNF is involved in hippocampal long-term depression and cell death mechanisms [29, 35, 46, 53]. Because PAI-1 is one of primary inhibitors of tPA, it was proposed that PAI-1 may be involved in the pathogenesis of MDD through inhibition of the proBDNF cleavage via its interaction with the tPA–plasminogen system.

Here, we investigated whether and how PAI-1 may contribute to MDD. PAI-1 knockout mice and tPA knockout mice were submitted to a range of behavioral tests that exhaustively assess depressive-like symptoms. Furthermore, we investigated the mechanisms relating PAI-1 to MDD using molecular, biochemical and pharmacological analyzes.

## Materials and methods

### Animals

Ten weeks old C57BL/129 PAI-1 knockout (PAI-1−/−) and C57BL/129 tPA knockout (tPA−/−) male mice and their corresponding wild-type littermates (PAI-1+/+; tPA+/+) were used in this study [6, 7]. The strain, number and age of mice used in each experiment are summarized in the Additional file 6: Table S1. All the experiments were approved by the French ministry of education and research (agreement numbers APAFIS#5115 and APAFIS#4359). The principal investigator (VA, personal license number 14–73) was accredited by the “Direction Départementale des Services Vétérinaires”. The animal investigations were performed under the current European directive (2010/63/EU) as incorporated in national legislation (Decree 87/848) and in authorized laboratories (GIP Cyceron; approval n° E14118001). Further details are described in the Additional file 1: Additional Materials and Methods.

### Assessment of depressive-like behaviors

We first set-up a depression screening system in mouse (*n* = 276; see Additional file 7: Table S2) to characterize the potential depressive-like phenotype of PAI-1 and tPA mice. To this aim, a range of behavioral tests were used for assaying each depression symptom: splash test, sucrose preference test, body weight, actimetry, rotarod, coat state, T-maze and forced swimming test. They are described in the Additional file 1: Additional Materials and Methods. Our methodological preclinical model thus consisted of exhaustively scoring primary (apathetic and anhedonic behaviors) and secondary (hypo- or hyperactivity, loss of motivation/effort, body weight, deficits of self-centered behaviors, cognitive deficits) depressive criteria (see Additional file 7: Table S2) by means of behavioral tests. One point was attributed when a significant difference was yielded between knockout and wild-type mice; the score was equal to 0 when there was no significant difference between groups. Thereafter, all the scores were added together forming a composite score. At the end, the depression-like phenotype was retained if and only if the composite score was at least equal to 4 points, including necessarily at least one of the two primary criteria.

### Perfusion and sampling

Animals were deeply anesthetized with isoflurane 5%, and thereafter maintained with 2.5% isoflurane in a 70%/30% mixture of NO_2_/O_2_. A transcardial perfusion was performed with ice cold 0.9% NaCl heparinized. Further details are described in the Additional file 1: Additional Materials and Methods.

### Protein extraction

Tissues were dissociated in ice-cold TNT buffer (50 mM Tris-HCl pH 7.4; 150 mM NaCl; 0.5% Triton X-100; Sigma-Aldrich, Saint-Quentin Fallavier, France), centrifuged for 20 min (12,000 g at 4 °C), then protein concentration was quantified using the BCA method (Pierce, Rockford, IL, USA).

### Fibrin-agar zymography assay

Proteins (20 μg) were electrophoresed in a 8% polyacrylamide gel under non reducing conditions. SDS was then exchanged with 2.5% Triton X-100 (Sigma-Aldrich). After washing off excess Triton X-100 with distilled water, the gel was carefully overlaid on a 1% agarose indicator support containing bovine fibrinogen, plasminogen and thrombin. Zymograms were then allowed to develop at 37 °C during several days and photographed at regular intervals using dark-ground illumination. Data were normalized to the mean value obtained for wild-type mice. Results shown are representative pictures of the zymograms and the corresponding quantifications made by calculating the area under the curve for each lysis band by using the ImageJ software (NIH).

### Immunoblotting

Proteins (20 μg) were resolved on 4–20% SDS-PAGE gel (Bio-rad, Marnes-la-Coquette, France) under denaturing conditions and transferred onto a polyvinylidene difluoride membrane. Membranes were blocked with Tris-buffered saline (TBS: 10 mM Tris and 200 mM NaCl, pH 7.4) containing 0.05% Tween-20 and 1% BSA. Blots were incubated overnight at 4 °C in blocking buffer with the primary antibody: rabbit anti-mBDNF (1/5000; ab108319; Abcam, Paris, France), rabbit anti-proBDNF (1/100; ab72440; Abcam) or rabbit anti-actin (1/1000; A2066; Sigma–Aldrich). Membranes were then incubated with peroxidase-conjugated anti-rabbit secondary antibodies (1/50000; Sigma-Aldrich). Proteins were visualized with an enhanced chemiluminescence western blot detection reagent (ECL Prime Western Blotting System; GE Healthcare; RPN2232) using ImageQuant LAS 4000 Camera Camera (GE Healthcare, Chicago, IL, USA).

### Ultra-high-pressure liquid chromatography coupled with tandem-mass spectrometry (UHPLC-MS/MS)

We developed and validated a novel UHPLC-MS/MS method for quantification of dopamine (DA), noradrenaline (NA) and serotonin (5-HT) in mice brain structures. Briefly, the method consisted in crushing in formic acid aqueous solution in acetonitrile containing acetic acid. The samples were then centrifuged and injected in the UHPLC-MS/MS system and quantification was obtained by a 1/X2 weighted calibration curves using the internal standard method. Lower limits of quantification in the injected solutions were 0.1 ng/mL for DA and 5-HT, and 0.5 ng/mL for NA. For further detail, see Additional file 1: Additional Materials and Methods and Additional file 8: Table S3.

### Drug administration

Escitalopram (ESC) and fluoxetine (FLX) were purchased from Carbosynth (Compton-Bershire, United Kingdom). Both drugs were dissolved in saline (NaCl 0.9%) and were administrated intraperitoneally (i.p.) at a concentration of 15 mg/kg (1 mg/ml) or 30 mg/kg (2 mg/ml) body weight. The drug dose was chosen based on previous preclinical studies [5, 11, 12, 17, 43]. Control groups received an equivalent volume of saline (vehicle: VEH). Mice were i.p. injected daily with ESC or FLX, or VEH, for 35 consecutive days. The behavioral tests were conducted from day 22 to day 35 of antidepressant treatment.

### Statistical analysis

Statistical analyses were performed using STATISTICA PRO® v13.0 software (StatSoft, Inc.). The distribution of samples was studied with Shapiro-Wilk’s tests. When data were normally distributed, Student’s *t*-tests were used. When analysis of the data sets via parametric approaches turned out to be inappropriate due to violation of residual normality, non-parametric approaches where used (Mann-Whitney’s *U*-tests for independent samples or Wilcoxon’s signed-rank tests for matched samples). An alpha level of *p* < 0.05 was used for determination of significance in all statistical tests; all tests are two tailed.

## Results

### PAI-1 deficiency results in a depressive-like phenotype in mice

**Fig. 1:**
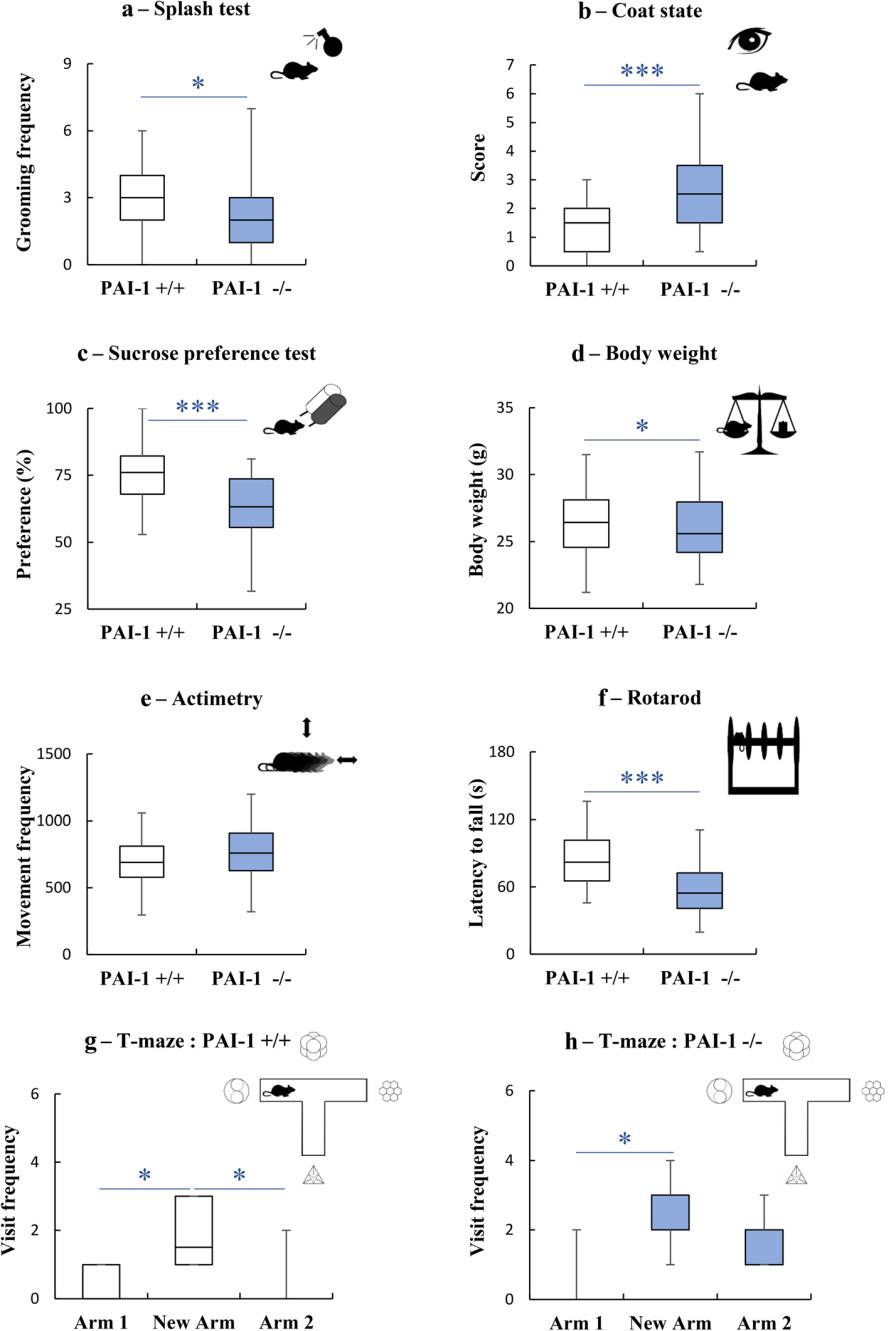
PAI-1 deficiency induces a depressive-like phenotype. Evaluation of the behavioral phenotype of PAI-1 knockout mice (PAI-1−/−) and of their wild-type littermates (PAI-1+/+). **a** Splash test: n_PAI-1+/+_ = 34; n_PAI-1 −/−_ = 41. **b** Coat state: n_PAI-1+/+_ = 35; n_PAI-1−/−_ = 42. **c** Sucrose preference test: n_PAI-1+/+_ = 35; n_PAI-1−/−_ = 39. **d** Body weight: n_PAI-1+/+_ = 15; n_PAI-1−/−_ = 15. **e** Actimetry: n_PAI-1+/+_ = 35; n_PAI-1−/−_ = 42. **f** Rotarod: n_PAI-1+/+_ = 33; n_PAI-1−/−_ = 40. **g**, **h** T-maze: n_PAI-1+/+_ = 12; n_PAI-1−/−_ = 9. Mann-Whitney U-tests (**a**, **b**), Student t tests (**c**-**f**), Wilcoxon signed-rank tests (**g**, **h**): *P < 0.05, **P < 0.01, ***P < 0.001. Boxplots show distributions with black horizontal lines indicating the median, box margins denoting the lower and upper quartiles. Whiskers show the minimum and maximum values

After setting up a depression screening system in mouse (see Additional file 7: Table S2), we have characterized the behavioral phenotype of PAI-1−/− and PAI-1+/+ mice. In the splash test, PAI-1−/− mice exhibited decreased frequency of grooming behavior when compared to PAI-1+/+ control mice (Fig. 1a; U = 506; *P* = 0.042), thus pointing to apathetic behavior in PAI-1−/− mice (Table 1; score = 1). A significant alteration of the coat state of PAI-1−/− mice was found (Fig. 1b; PAI-1−/−: 2.58; PAI-1+/+: 1.27; U = 357.5, *P* = 0.00007) indicating a deficit of self-centered behaviors (Table 1; score = 1). PAI-1-deficient mice also decreased preference for sucrose containing water compared to PAI-1+/+ mice (Fig. 1c; t = 4.08, *P* = 0.00011), demonstrating an anhedonic behavior in PAI-1−/− mice (Table 1; score = 1). In addition, there was a significant difference in the body weight between the two genotypes (Fig. 1d; t = 2.08, *P* = 0.047; 24.12 g ± 1.60 for PAI−/− mice versus 25.54 g ± 2.11 for PAI-1+/+ mice) (Table 1; score = 1). In terms of actimetry, both groups of mice showed a similar locomotor activity (Fig. 1e; PAI-1−/−: number of movements = 746 ± 211; PAI-1+/+: number of movements = 694 ± 191; t = − 1.13, *P* = 0.261). In contrast, PAI-1−/− mice exhibited altered rotarod performance; indeed, latencies to fall were 57.35 s and 84.51 s for knockout and wild-type mice, respectively (Fig. 1f; t = 4.83, *P* = 0.00001). These results suggest impairment in motivation/effort in PAI-1 knockout mice (Table 1; score = 1). Finally, spatial cognitive abilities were examined by using a place recognition task in a T-maze which required the use of room cues. During the test phase, PAI-1+/+ mice discriminated correctly the newly opened arm from the familiar ones since they visited significantly more often the new arm than arms 1 and 2 (Fig. 1g; Arm 1 vs New arm: Z = 2.37, *P* = 0.018; Arm 2 vs New arm: Z = 2.10 *P* = 0.036), therefore indicating good spatial performance in control mice. PAI-1−/− mice entered more often in the new arm relative to familiar arm 1 (Fig. 1h; Arm 1 vs New arm: Z = 2.37, P = 0.018). However, the number of visits of the familiar arm 2 was not different from the newly open arm (Fig. 1h; Z = 1.68, *P* = 0.093). These observations reveal that the deficiency of PAI-1 alters the ability of mice to orientate properly (Table 1; score = 1). Overall, considering all behavioral procedures described above, PAI-1−/− mice obtained a composite score of 6 points, which is equivalent to six depressive-related symptoms of the screening system, including apathetic and anhedonic behaviors, two hallmarks of MDD in humans (Table 1, column 2; see Additional file 7: Table S2). Conversely, PAI+/+ mice showed any symptom. Altogether, these results demonstrate a depressive-like phenotype in PAI-1−/− mice. Interestingly, this deficit observed in PAI-1 −/− mice cannot be attributed to any brain weight or volume disparity (see Additional file 2: Figure S1; see Additional file 1: Additional Materials and Methods).

**Table 1:** Depressive-like phenotype in mice. Relative score of PAI-1 knockout mice and tPA knockout mice

Depressive-like behaviors in mice		PAI-1−/−	tPA−/−
Apathetic behavior	Splash test	1	0
Anhedonic behavior	Sucrose preference test	1	0
Body weight	Balance	1	1
Hypoactivity or hyperactivity	Actimetry	0	0
Loss of motivation/effort	Rotarod	1	0
Deficits of self-centered behaviors	Coat state	1	1
Cognitive deficits	T-maze	1	1
Composite Score		6	3

*PAI-1−/−* PAI-1 knockout mice, *tPA−/−* tPA knockout mice

### The depressive-like phenotype of PAI-1−/− mice is independent of the tPA-BDNF axis

To determine whether the involvement of PAI-1 in depression was associated, or not, with its interaction with tPA, we also examined the behavioral phenotype of tPA−/− mice and that of their wild-type littermates with our depression screening system (see Additional file 7: Table S2; see Additional file 3: Figure S2). At the end of the behavioral tests, tPA knockout mice reached a composite score of 3 (Table 1, column 3). Importantly, they did not exhibit apathetic or anhedonic behavior. Thus, deficiency in tPA does not influence the depressive-like phenotype of mice compared to their wild-type littermates. This first set of behavioral tests suggests that the depressive-like phenotype observed in PAI-1−/− mice is tPA-independent.

**Fig. 2:**
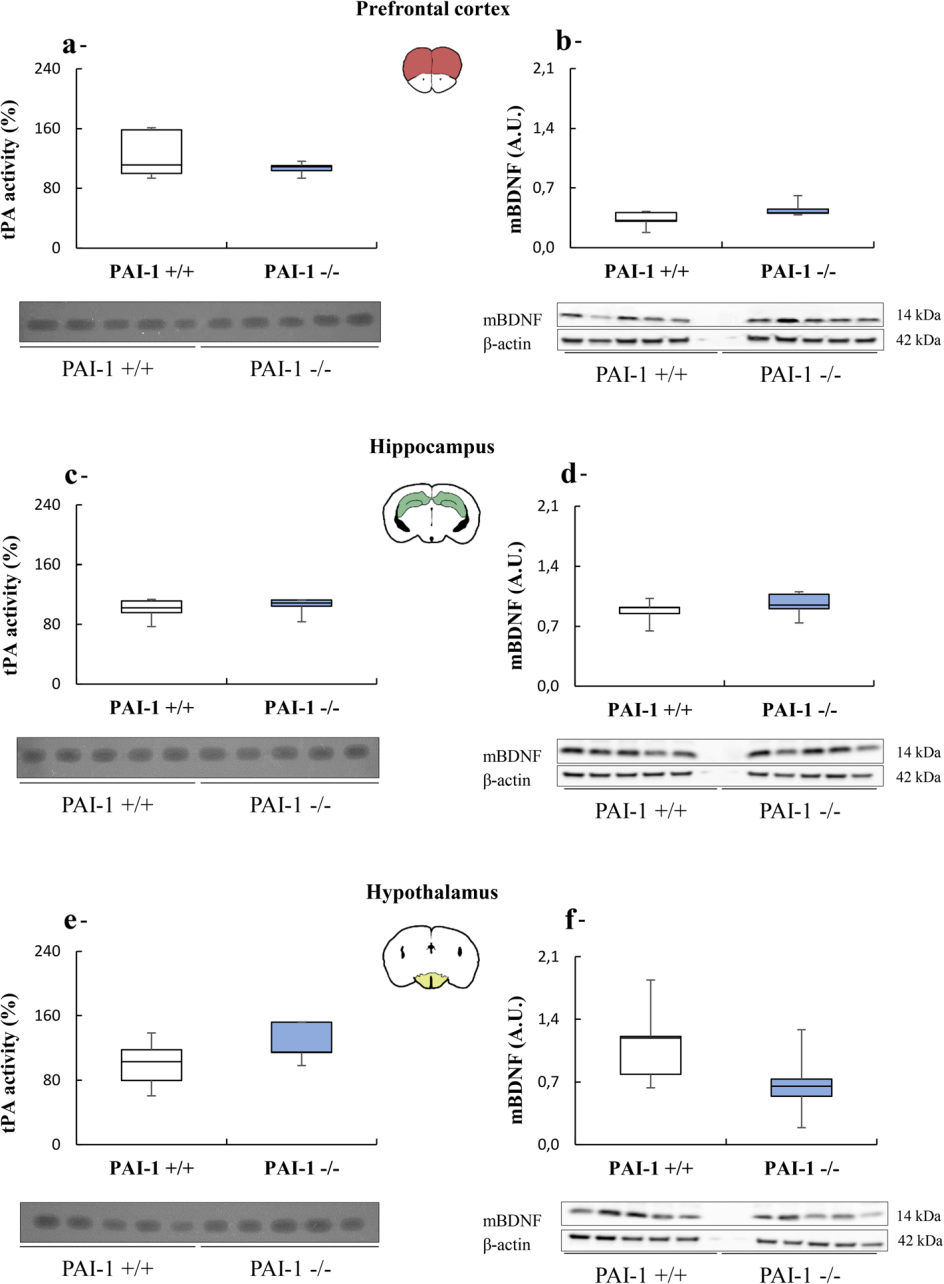
Influence of PAI-1 deficiency on depressive-like phenotype is independent of tPA and mBDNF. Fibrin-agar zymography assays (tPA proteolytic activity) and immunoblots (mBDNF levels) in the prefrontal cortex (**a**, **b**), hippocampus (**c**, **d**), and hypothalamus (**e**, **f**) of PAI-1 knockout mice (PAI-1−/−) and of their wild-type littermates (PAI-1+/+). *n* = 5 for each structure / genotype. Student t tests: *P* > 0.05. Boxplots show distributions with black horizontal lines indicating the median, box margins denoting the lower and upper quartiles. Whiskers show the minimum and maximum values

The next step of our study examined cerebral tPA and BDNF levels in several brain structures related to depression (prefrontal cortex, hippocampus, hypothalamus) in PAI-1−/− and PAI-1+/+ mice. tPA activity was similar in both groups of mice whatever the brain areas (Fig. 2a: t = − 0.12, *P* = 0.907; Fig. 2c: t = − 0.52, *P* = 0.618; Fig. 2e: t = − 1.51, *P* = 0.170). These data argue for a role of PAI-1 in depression-like behaviors by a mechanism independent of its role of tPA inhibitor. To ascertain this claim, we measured the levels of mBDNF in the same areas of PAI-1−/− and PAI-1+/+ mice. Our results revealed that the neurotrophin levels did not differ between groups (Fig. 2b: t = − 2.04, *P* = 0.076; Fig. 2d: t = − 0.91, *P* = 0.387; Fig. 2f: t = 1.65, *P* = 0.138). These data thus confirm the role of PAI-1 in depression, by a mechanism independent of the tPA–plasminogen–BDNF axis.

### PAI-1 deficiency is associated with reduced levels of parenchymal 5-HT and DA

**Fig. 3:**
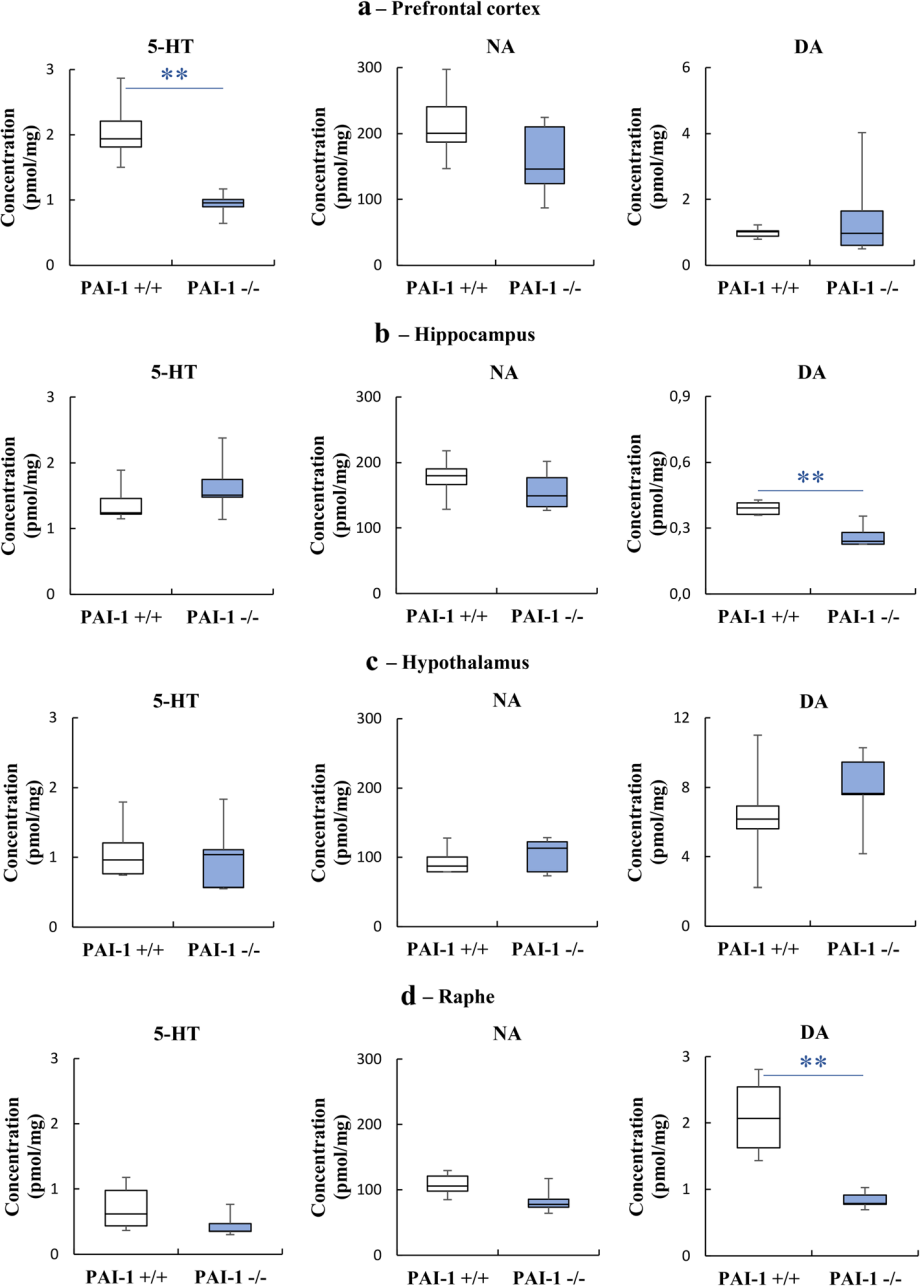
PAI-1 deficiency is associated with reduced levels of serotonin and dopamine. Concentrations of serotonin (5-HT), noradrenalin (NA) and dopamine (DA) in PAI-1 knockout mice (PAI-1−/−) and their wild-type littermates (PAI-1+/+). Ultra-high-pressure liquid chromatography coupled with tandem-mass spectrometry was performed in the prefrontal cortex (**a**), hippocampus (**b**), hypothalamus (**c**) and dorsal raphe (**d**). *n* = 5 for each structure / genotype. Mann-Whitney U-tests: **P < 0.01. Boxplots show distributions with black horizontal lines indicating the median, box margins denoting the lower and upper quartiles. Whiskers show the minimum and maximum values

Given the monoaminergic hypothesis of MDD, we measured the levels of 5HT, NA and DA in brain structures (prefrontal cortex, hippocampus, hypothalamus, dorsal raphe) in PAI-1−/− and PAI-1+/+ mice. Our data demonstrate a significant decrease of 5-HT levels in prefrontal cortex of PAI-1 knockout mice when compared with those of PAI-1+/+ mice (Fig. 3a; U = 0, *P* = 0.008). In hippocampus and dorsal raphe, the levels of DA were also significantly reduced in PAI-1−/− mice when compared to control mice (Fig. 3b, d; Hippocampus: U = 0, P = 0.008; Raphe: U = 0, P = 0.008). The levels of NA were not affected by PAI-1 deficiency whatever the brain area (Fig. 3a: U = 6, *P* = 0.222; Fig. 3b: U = 8, *P* = 0.421; Fig. 3c: U = 10, *P* = 0.690; Fig. 3d: U = 4; *P* = 0.095). Altogether, these data reveal a strong relationship between the amounts of 5-HT and DA present in specific cerebral structures and the expression of PAI-1.

### PAI-1 is necessary to mediate the antidepressant effects of SSRIs

**Fig. 4:**
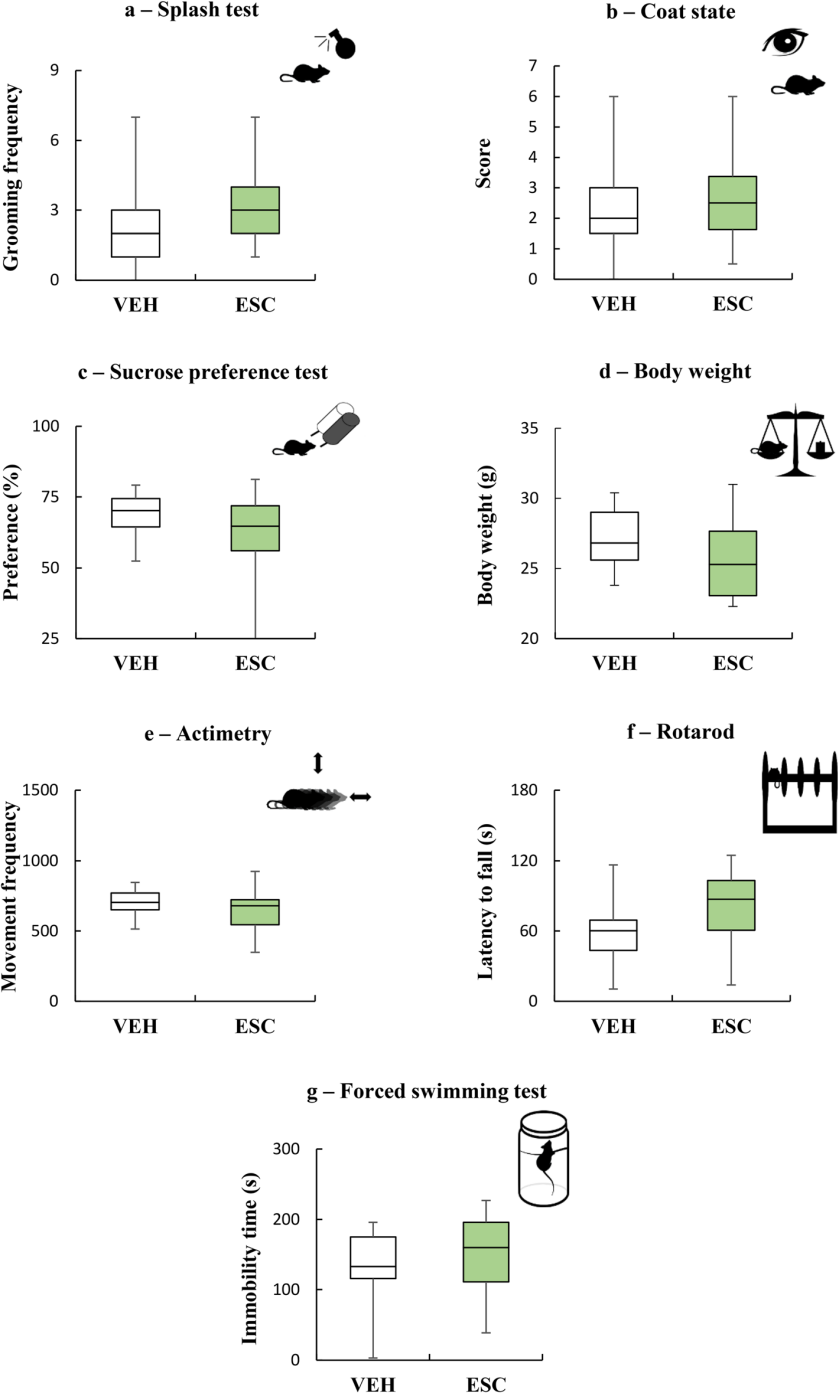
PAI-1 knockout mice fail to respond to escitalopram treatment. Evaluation of the behavioral phenotype of PAI-1 knockout mice submitted to an escitalopram chronic treatment (35 days) at 15 mg/kg. Escitalopram: ESC; Vehicle (NaCl 0.9%): VEH. **a** Splash test: n_VEH_ = 17; n_ESC_ = 18. **b** Coat state: n_VEH_ = 17; n_ESC_ = 18. **c** Sucrose preference test: n_VEH_ = 17; n_ESC_ = 18. **d** Body weight: n_VEH_ = 17; n_ESC_ = 18. **e** Actimetry: n_VEH_ = 17; n_ESC_ = 17. **f** Rotarod: n_VEH_ = 11; n_ESC_ = 12. **g** Forced swimming test: n_VEH_ = 17; n_ESC_ = 17. Mann-Whitney U-tests (**a**, **c**, **d**, **g**), Student t tests (**b**, **e**, **f**): *P* > 0.05. Boxplots show distributions with black horizontal lines indicating the median, box margins denoting the lower and upper quartiles. Whiskers show the minimum and maximum values

Because SSRIs are the most frequently prescribed antidepressants [41], we tested the effects of treatment with escitalopram, or fluoxetine, in PAI-1−/− mice. For this pharmacological study, we replaced the cognitive task of our screening behavioral tests by the forced swimming test (FST) as it is the gold standard assay for screening of antidepressant drugs. We first applied an escitalopram chronic treatment (35 days) at 15 mg/kg. The data showed that none of the depressive-like symptoms was counterbalanced by the escitalopram treatment in PAI-1−/− mice (Fig. 4a: U = 114.5, *P* = 0.207; Fig. 4b: t = − 0.99, *P* = 0.328; Fig. 4c: U = 105, *P* = 0.118; Fig. 4d: U = 100, *P* = 0.083; Fig. 4e: t = − 1.43, *P* = 0.161; Fig. 4f: t = − 1.36, *P* = 0.186). This was also true for the FST since the duration of immobility was similar for the two groups (Fig. 4g: U = 112, *P* = 0.274). We then examined the effect of a higher dose of the same antidepressant, i.e. 30 mg/kg i.p. This second experiment confirmed our previous observation that chronic treatment with escitalopram did not exert antidepressant-like effect in PAI-1−/− mice, whatever the dose administered or the assessed symptom (see Additional file 4: Figure S3a: U = 65, *P* = 0.712; see Additional file 4: Figure S3b: t = 1.11, *P* = 0.281; see Additional file 4: Figure S3c: U = 56, *P* = 0.378; see Additional file 4: Figure S3d: t = − 0.86, *P* = 0.397; see Additional file 4: Figure S3e: U = 58, *P* = 0.443; see Additional file 4: Figure S3f: t = − 0.92, *P* = 0.369; see Additional file 4: Figure S3 g: t = 0.96, *P* = 0.348). To further understand whether this lack of efficacy was specific to escitalopram or generalized to other SSRIs, we subjected PAI-1−/− mice to treatment with fluoxetine, another SSRI largely used in clinic. Our data once again demonstrated the ineffectiveness of the antidepressant to reverse depressive symptoms of PAI-1−/− mice (see Additional file 5: Figure S4a: U = 68.5, *P* = 0.611; see Additional file 5: Figure S4b: U = 77.5, *P* = 0.979; see Additional file 5: Figure S4c: U = 53, *P* = 0.303; see Additional file 5: Figure S4d: t = − 1.28, *P* = 0.214; see Additional file 5: Figure S4e: t = − 1.16, *P* = 0.259; see Additional file 5: Figure S4f: t = 1.86, *P* = 0.078; see Additional file 5: Figure S4 g: U = 47.5; *P* = 0.160).

## Discussion

In the present study, we unveil an original genetic model of depression namely the deletion of PAI-1. By using a depression screening system adapted to mouse, we demonstrate that the deficiency of PAI-1 clearly results in a depressive-like phenotype. In accordance with the monoamine hypothesis of depression, we demonstrate that these phenotypic characteristics are associated with changes in monoamine levels in brain structures known to be involved in MDD. We then establish that the involvement of PAI-1 in depression is independent of the tPA-BDNF axis. Finally, because of the inefficacy of escitalopram and fluoxetine treatments, we authenticate the PAI-1−/− mice as a resistant-SSRI genetic model of depression.

The diagnosis of MDD in humans is established by the presence of at least five symptoms out of a list of nine, which must include depressed mood and / or anhedonia [2, 49]. In our study, we chose to elaborate a depression screening system based on a large battery of behavioral tests since all data obtained can be combined to create a composite score which offers a more complete evaluation of the phenotype profile. The evaluation of PAI-1−/− mice showed the presence of six symptoms out of a list of seven in our behavioral screening system: anhedonia, apathy, weight loss, impairment in motivation/effort, self-centered behaviors deficit and cognitive dysfunction. Observed symptoms are multiple and in addition, they include mandatory primary criteria like in humans, providing an excellent face validity of the PAI-1 knockout mouse model.

To further explore the PAI-1−/− mouse model, we examined whether the depression-related behaviors of PAI-1−/− mice may be linked to monoamine transmission alterations. We observed a decrease of the concentrations of 5-HT and DA, two neurotransmitters identified for their involvement in MDD. Interestingly, this depletion in monoamine levels was found specifically in the prefrontal cortex, hippocampus, and dorsal raphe, three brain regions known to play a crucial role in psychic disorders [10, 21, 25, 28, 33, 39], including MDD. In this respect, the monoamines changes in PAI-1−/− mice demonstrate the construct validity of this animal model of depression. Electrochemical assays or microdialysis techniques would be relevant to measure the synaptic concentrations of neurotransmitters and thus to better understand the mechanisms underlying the role of PAI-1 in MDD.

We then examined the response of PAI-1−/− mice to antidepressant drugs. Chronic administration of escitalopram or fluoxetine did not reverse depressive-like behaviors. The inefficacy of treatments in PAI-1−/− mice strongly suggests that the SSRIs mechanism of action is directly dependent of PAI-1. Our hypothesis is strengthened by Tsai et al.’ study [48] demonstrating that the genetic variants of *SERPINE1* gene in humans decrease the acute therapeutic response to SSRIs. Moreover, the present results confirm the assumption that antidepressants are less effective in conditions where vulnerability to depression, because of the presence of genetic, personality or developmental risks factor, is elevated [51, 52]. Consequently, the PAI-1−/− mouse represents an excellent model of predisposition to depression for the characterization of future drug candidates for patients not responsive to current treatments.

Several authors have suggested that PAI-1 could be involved in the pathogenesis of MDD through inhibition of the cleavage of the proBDNF into mBDNF via its blockage of the proteolytic activity of tPA. Here, we demonstrate that the depressive-like phenotype induced by deficiency of PAI-1 is not dependent on the tPA–BDNF axis. First, tPA knockout mice do not exhibit depressive-like behaviors. Second, no variation of active tPA or mBDNF levels was observed in PAI-1-depressed mice relative to controls. It is possible to hypothesize that PAI-1 would act independently of tPA, as previously demonstrated for its ability to promote cell survival and neuronal network [42]. This question should be examined in more details in futures studies. Interestingly, tPA-deficient mice have been described to display behavioral disinhibition associated with a decrease of serotonin levels specifically in thalamus and caudate putamen [38]. These data are in direct line with ours, unequivocally demonstrating that cerebral selective impairments of the serotonin circuitry, mediated by tPA or PAI-1, predispose to specific and independent forms of emotional impairment.

Many clinical studies have shown elevated plasma PAI-1 levels in depressed patients [14, 22, 24]. In addition, a number of *SERPINE1* genetic variants have been associated with MDD [48]. At the preclinical scale, increased cerebral and vascular PAI-1 concentrations have been found in rodents subjected to a CUMS procedure [20, 45]. Corticosterone also markedly raises the levels of blood PAI-1 in mice [44]. One can postulate that PAI-1 could be involved in the negative feedback loop of the hypothalamic-pituitary-adrenal axis. In the absence of PAI-1 (PAI-1 knockout mice), the negative feedback does not lead to an elevated and unregulated cortisolemia, likely cause of depression pathogenesis. The role played by PAI-1 in MDD should be addressed with more details in future studies thanks to the development of new tools such as the generation of conditional PAI-1 knock-out mice.

## Conclusions

In summary, we provide here a new genetic model of depression, the PAI-1 knockout mouse, with strong face and construct validity. This preclinical model being in direct line with genetic variants of *SERPINE1* gene observed in humans strengthens the fact that PAI-1 is a factor of predisposition to MDD. In addition, the PAI-1 knockout mouse is also a model of resistance to antidepressants such as SSRIs. Finally, this study provides the first demonstration of the involvement of PAI-1 in depression by a mechanism independent of BDNF, and suggests that PAI-1 could be an innovative target for the development of new drugs for MDD. Further investigations will be required to understand how the signaling cascades downstream of PAI-1 affect the risk of vulnerability to depression and engage the mental disorder.

## Supplementary information

**Additional file 1.** Additional Materials and Methods. Animals. Assessment of depressive-like behaviors (Splash test; Sucrose preference test; Body weight; Actimetry; Rotarod; Coat state; T-maze; Forced swimming test). Perfusion and sampling. Ultra-high-pressure liquid chromatography coupled with tandem-mass spectrometry (Analytical Methods; Quantification). Magnetic Resonance Imaging.

**Additional file 2: Figure S1.** The depressive-like phenotype of PAI-1 −/− mice is not attributed to any brain weight or volume disparity. Quantitative analyses of brain volume (a) and weight (b) in PAI-1 knockout mice (PAI-1 −/−) and of their wild-type littermates (PAI-1 +/+). (c) Representative T2-weighted images of PAI-1 −/− and PAI-1 +/+ mice’s brain. *n* = 3 for each group. Mann-Whitney U-tests: *P* > 0.05. Boxplots show distributions with black horizontal lines indicating the median, box margins denoting the lower and upper quartiles. Whiskers show the minimum and maximum values.

**Additional file 3: Figure S2.** tPA deficiency does not induce a depressive-like phenotype. Evaluation of the behavioral phenotype of tPA knockout mice (tPA−/−) and of their wild-type littermates (tPA+/+). (a) Splash test: n_tPA+/+_ = 20; n_tPA−/−_ = 20. (b) Coat state: n_tPA+/+_ = 20; n_tPA−/−_ = 20. (c) Sucrose preference test: n_tPA+/+ EE_ = 20; n_tPA−/− EE_ = 20. (d) Body weight: n_tPA+/+ EE_ = 15; n_tPA−/−_ = 15. (e) Actimetry: n_tPA+/+_ = 19; n_tPA−/−_ = 20. (f) Rotarod: n_tPA+/+_ = 20; n_tPA−/−_ = 20. (g-h) T-maze: n_tPA+/+_ = 15; n_tPA−/−_ = 13. Mann-Whitney U-tests (a-b), Student t tests (c-f), Wilcoxon signed-rank tests (g-h): *P < 0.05. Boxplots show distributions with black horizontal lines indicating the median, box margins denoting the lower and upper quartiles. Whiskers show the minimum and maximum values.

**Additional file 4: Figure S3.** PAI-1 knockout mice fail to respond to escitalopram treatment. Evaluation of the behavioral phenotype of PAI-1 knockout mice (PAI-1−/−) submitted to an escitalopram chronic treatment (35 days) at 30 mg/kg. Escitalopram: ESC; Vehicle (NaCl 0.9%): VEH. (a) Splash test: n_VEH_ = 12; n_ESC_ = 12. (b) Coat state: n_VEH_ = 12; n_ESC_ = 12. (c) Sucrose preference test: n_VEH_ = 12; n_ESC_ = 12. (d) Body weight: n_VEH_ = 12; n_ESC_ = 12. (e) Actimetry: n_VEH_ = 12; n_ESC_ = 12. (f) Rotarod: n_VEH_ = 12; n_ESC_ = 12. (g) Forced swimming test: n_VEH_ = 12; n_ESC_ = 11. Mann-Whitney U-tests (a, c, e), Student t tests (b, d, f, g): P > 0.05. Boxplots show distributions with black horizontal lines indicating the median, box margins denoting the lower and upper quartiles. Whiskers show the minimum and maximum values.

**Additional file 5: Figure S4.** PAI-1 knockout mice fail to respond to fluoxetine treatment. Evaluation of the behavioral phenotype of PAI-1 knockout mice (PAI-1−/−) submitted to a fluoxetine chronic treatment (35 days) at 15 mg/kg. Fluoxetine: FLX; Vehicle (NaCl 0.9%): VEH. (a) Splash test: n_VEH_ = 12; n_FLX_ = 13. (b) Coat state: n_VEH_ = 12; n_FLX_ = 13. (c) Sucrose preference test: n_VEH_ = 11; n_FLX_ = 13. (d) Body weight: n_VEH_ = 12; n_FLX_ = 13. (e) Actimetry: n_VEH_ = 12; n_FLX_ = 13. (f) Rotarod: n_VEH_ = 10; n_FLX_ = 11. (g) Forced swimming test: n_VEH_ = 12; n_FLX_ = 12. Mann-Whitney U-tests (a-c, g), Student t tests (d-f): P > 0.05. Boxplots show distributions with black horizontal lines indicating the median, box margins denoting the lower and upper quartiles. Whiskers show the minimum and maximum values.

**Additional file 6: Table S1.** Number of mice used in the study.

**Additional file 7: Table S2.** Quantification modeling of depressive-like behaviors in mouse.

**Additional file 8: Table S3.** Multiple Reaction Monitoring (MRM) method parameters. The MRM product ions in brackets are uses as confirmation transitions.

## Data Availability

The datasets used and/or analyzed during the current study are available from the corresponding author on reasonable request.

## Abbreviations

5-HMT: 5-hydroxy-N-*ω*-methyltryptamine
5-HT: Serotonin
BDNF: Brain-derived neurotrophic factor
CUMS: Chronic unpredictable mild stress
DA: Dopamine
DHBA: 3,4-dihydroxybenzylamine
ESC: Escitalopram
FLX: Fluoxetine
FST: Forced swimming test
MDD: Major depressive disorder
MDE: Major depressive episodes
MRM: Multiple reaction monitoring
NA: Noradrenaline
PAI-1: Plasminogen activator inhibitor-1
SSRIs: Selective serotonin reuptake inhibitors
tPA: Tissue-type plasminogen activator
UHPLC-MS/MS: Ultra-high-pressure liquid chromatography coupled with tandem-mass spectrometry
VEH: Vehicle
