# Supplementary material for: Plasminogen Activator Inhibitor-1 (PAI-1) deficiency predisposes to depression and resistance to treatments
Source: Acta Neuropathol Commun. 2019 Oct 14;7:153. doi: 10.1186/s40478-019-0807-2 (PMC6791031; doi:10.1186/s40478-019-0807-2)
Supplement: Supplementary file 1 — Additional file 1. Additional Materials and Methods. Animals. Assessment of depressive-like behaviors (Splash test; Sucrose preference test; Body weight; Actimetry; Rotarod; Coat state; T-maze; Forced swimming test). Perfusion and sampling. Ultra-high-pressure liquid chromatography coupled with tandem-mass spectrometry (Analytical Methods; Quantification). Magnetic Resonance Imaging. [file 40478_2019_807_MOESM1_ESM.docx]

**ADDITIONAL MATERIALS AND METHODS**

**Animals**

The mice were housed in standard polypropylene cages (22 x 37 x 19 cm, Charles River, L’Arbresle, France; 5 mice per cage) containing nesting material and cardboard tubes. These housing conditions were designed to meet animal welfare and to allow for the expression of natural behaviors. The mice were maintained in a temperature and humidity controlled room (12-hour light/dark inverted cycle) and had free access to water and food (Animal care facilities: Centre Universitaire de Ressources Biologiques (CURB), Caen Normandy University, France; approval n° A14118015). All the experiments were performed between 8AM-5PM in a room with dim illumination (6 lx). The strain, number and age of mice used in each experiment are summarized in the Additional file 6: Table S1. All behavioral tests were performed by an investigator blinded of the pharmacological treatment.

**Assessment of depressive-like behaviors**

*Splash test*- was performed in the mouse home cage. The test consisted of squirting two sprays of an atomizer containing 10% sucrose solution on the dorsal coat of the mouse. Because of its viscosity, the sucrose solution induces grooming behavior. After a 20-sec delay, the frequency of grooming behavior was recorded for a period of 5 min [9, 10, 13]. The splash test evaluates the apathetic behavior.

*Sucrose preference test*- was performed with individually housed mice for 24 h. Mice were given a free choice between two bottles, one with 10% sucrose solution and another with tap water. To prevent possible effects of side preference in drinking behavior, the position of the bottles was switched between cages. After the test, the consumption of water and sucrose solution was estimated by weighing the bottles. The preference for sucrose was calculated as a percentage of consumed sucrose solution of the total amount of liquid drunk [19, 20]. A flow sucrose preference is considered to indicate anhedonic behavior.

*Body weight*- was measured using a precision weighing scale (Kern PCB, Germany) before each behavioral evaluation. For the experiments with the antidepressants, body weight was measured daily.

*Actimetry*- Spontaneous locomotor activity was quantified by using a rack of eight activity cages equipped with horizontal infrared beams located across the long axis of the cage (Imetronic®, Pessac, France). Mice were placed in individual acrylic chambers (30 x 20 x 20 cm) for 30 min. The number of horizontal movements was determined by breaks in movement-sensitive photo-beams that were then converted into locomotor activity counts [6]. This test evaluates the hypo- or hyperactivity.

*Rotarod*- consisted of a black striated rod separated in five compartments situated 18 cm high above four tilting planks (Letica LE8500, Bioseb®, Vitrolles, France). 24 h before the test, mice were trained to the task for three trials (intertrial interval of 15 min) using the following strategy: the rod accelerated from 4 to 40 rpm over 5 min [12, 17, 18]. The time before falling was used as an index of loss of motivation/effort.

*Coat state*- consisted on the assessment of fur state on seven parts of the body: head, neck, forepaws, back, abdomen, hindpaws, tail [4, 13]. Each zone was scored 0 if in a good state (the fur is smooth and shiny, with no tousled, spiky patches), 0.5 if in moderately bad state (the fur is slightly fluffy with some spiky patches), and 1 in bad state (the fur is dirty and fluffy on most of the body with slight staining). The sum of these scores is an indicator of self-centered behaviors.

*T-maze*- To assess cognitive functions, we used a two-trial place recognition task previously developed to study spatial cognition processes in rats and mice [5, 7, 8, 14, 15]. The standard T-maze used in this study was constructed of white plastic with three identical arms (31 x 8 x 15 cm; BMP Chaudronnerie, Bretteville-sur-Odon, France). Extra-maze distal cues were suspended on the black curtains of the room walls at a distance of 123 cm from the maze (from the top of the cues to the center of the maze). The behavioral procedure consisted of two trials separated by a 2.5-h inter-trial interval. During the acquisition phase (trial 1), one randomly-chosen arm of the T-maze was closed with a guillotine door. Each mouse was then placed in the start arm (Arm 1), the head facing away from the center of the maze, and allowed to freely visit the accessible parts of the maze (Arm 1/Arm 2) for 5 min. At the end of the trial, the labeled mouse was then replaced in its home cage. During the test phase (trial 2), animals had free access to all three arms (Arm 1/Arm 2/New arm) for 1 min. Spatial performance was assessed through the comparison of the number of visits made in each arm (considered only when the mouse passed two-thirds of the arm). It has been shown that control mice spend more time in the New arm than in the familiar arms (Arm 1/Arm2) therefore indicating processing of remote spatial cues.

*Forced swimming test (FST)*- This test is the gold standard to screening for antidepressant drugs and thus was conducted here in pharmacological experiments [1–3, 11, 16]. Mice were gently placed in a glass cylinder (9 cm in diameter, 30 cm high) filled with 20 cm of water (±25°C) for a single 6-min exposure. After intense escape-directed behavior (i.e. swimming, climbing), animals stop struggling and show passive immobile behavior. Duration of immobility was recorded during the last 4 min of the test. This test is believed to reflect behavioral despair.

**Perfusion and sampling**

Brains were removed and cut in 1 mm thick slices using a brain matrix (Delta Microscopies, Ayguesvives, France). Sections were kept on ice in a cold saline solution and structures of interest (prefrontal cortex, hippocampus, hypothalamus, raphe) were then sampled using a biopsy punch (Delta Microscopies, Ayguesvives, France). Sections were then stored at −80°C immediately after collection until processing.

**Ultra-high-pressure liquid chromatography coupled with tandem-mass spectrometry**

*Analytical Methods*

Preparation consisted in weighting the brain structures precisely (between 3 and 20 mg) and adding 1 µL of a solution containing internal standards (5 µg/mL and 50 ng/mL of 3,4-dihydroxybenzylamine (DHBA) and 5-hydroxy-N-*ω*-methyltryptamine (5-HMT), respectively) per mg of brain. The mixture was stored for 30 minutes in ice in obscurity. Then the volume was completed with a solution of 2% (w/v) formic acid in water to 100 µL if the mass of sample was smaller or equal to 10 mg, for higher brain mass 150 µL 2% (w/v) formic acid will be added. The samples were then centrifuged for 5 minutes at 10 000 g at +4°C and a volume of 7 µL of the supernatant was injected in the UHPLC-MS/MS system.

Analyzes were carried out on UHPLC Nexera X2 system consisting of two binary pumps with an auto-sampler and a column oven (Shimadzu, Kyoto, Japan). The mobile phase, composed of 0.1% (v/v) aqueous acetic acid (solvent A) and acetonitrile (solvent B), was delivered at a flow rate of 0.6 mL/min under the following gradient conditions: starting from 5% B to 1.5 min and then increasing to 27.5% B in 1.5 min. The column was then equilibrated by holding the initial conditions for 2 min. UHPLC was carried out on a Kinetex biphenyl column (100 mm x 2.1 mm i.d., 1.7 µm particle diameter) from Phenomenex (Torrance, CA, USA). The temperature was fixed at 30°C, the column was equilibrated by holding the initial conditions for 2 min.

Mass spectrometry was used for detection of compounds. The UHPLC system was interfaced with an electrospray triple quadrupole mass spectrometer (LCMS 8030 Plus; Shimadzu). The mass spectrometer was used in the Multiple Reaction Monitoring (MRM) acquisition mode after positive ESI except for 3,4-dihydroxy-phenyl acetic acid using negative ESI. LabSolutions 5.86 SP1 software was used to process the data. The measurements were performed at 250°C desolvation temperature, 300°C source temperature, 2.5 L/min cone gas (N2) and 15 L/min desolvation gas (N2). The capillary voltage was +4.5 kV. The mass spectrometer was programmed to allow the [M+H]+ or [M-H]- ions of monoamines to pass through the first quadrupole (Q1) into the collision cell (Q2). The product ions were monitored through the third quadrupole (Q3) for each compound, two masses were selected, the first one for quantification and the second one for confirmation of the compound according to European Commission Decision 2002/657/EC. The transitions used for each compound were summarized in the Additional file 8: Table S3.

*Quantification*

The limits of quantification were considered as the lowest concentrations of the calibration curve. Two internal standards were used. The first one was 5-HMT which was used for the quantification of 5-HT. The second one was DHBA used for the quantification of DA and NA (see Additional file 8: Table S3). Calibrators were prepared in water at the following concentrations: 0.1, 0.5, 1, 2, 5, 10, 20, 50, 100, 500, 750 and 1000 ng/mL; quality control samples were prepared at 30, 400 and 800 ng/mL. A weighting of 1/X^2^ was used for all the calibration-curve fittings between 0.1 and 1000 ng/mL for DA and 5-HT, and between 0.5 and 1000 ng/mL for NA. Quality controls were added in the injection sequence each 10 samples to check a ±20% accuracy target. Recoveries were calculated by the ratio of signals obtained for samples spiked before and after extraction. For all compounds recoveries were higher than 88% (n=5 for each compound). Intra-batch and inter-batch precisions for all compounds were lower than 10.0% and 12.5%, respectively (n=9 for each compound). Accuracy was evaluated for each compound between -8% and +10.3% (n=10 for each compound). This highly sensitive, specific and accurate UHPLC-MS/MS method provides acceptable performance for quantifying simultaneously DA, NA and 5-HT in the mouse brain.

**Magnetic Resonance Imaging**

PAI-1 +/+ and PAI-1 -/- mice were anesthetized with isoﬂurane 2% in a 70%/30% mixture of NO_2_/O_2_ via a nose cone with respiratory monitoring. Body temperature was maintained at ˷ 36.5°C by circulating warm water in a heating pad. Magnetic resonance imaging (MRI) was performed on a 7.0T Pharmascan MRI system (Bruker, Germany) equipped with surface coils. T2‐weighted images were acquired by use of a multislice multiecho sequence: echo time (TE)/repetition time (TR), 50/8,6 ms; and spatial resolution, 70 x 70 x 1500 µm, slice thickness: 0,15 mm, slices: 96, acquisition time: 17m 26s 400ms.

**REFERENCES**

1. Bogdanova OV, Kanekar S, D’Anci KE, Renshaw PF (2013) Factors influencing behavior in the forced swim test. Physiol Behav 118:227–239. doi: 10.1016/j.physbeh.2013.05.012

2. Can A, Dao DT, Arad M, Terrillion CE, Piantadosi SC, Gould TD (2012) The mouse forced swim test. J Vis Exp JoVE e3638. doi: 10.3791/3638

3. Castagné V, Moser P, Roux S, Porsolt RD (2010) Rodent models of depression: forced swim and tail suspension behavioral despair tests in rats and mice. Curr Protoc Pharmacol Chapter 5:Unit 5.8. doi: 10.1002/0471141755.ph0508s49

4. Culig L, Surget A, Bourdey M, Khemissi W, Le Guisquet A-M, Vogel E, et al. (2017) Increasing adult hippocampal neurogenesis in mice after exposure to unpredictable chronic mild stress may counteract some of the effects of stress. Neuropharmacology 126:179–189. doi: 10.1016/j.neuropharm.2017.09.009

5. Dellu F, Contarino A, Simon H, Koob GF, Gold LH (2000) Genetic differences in response to novelty and spatial memory using a two-trial recognition task in mice. Neurobiol Learn Mem 73:31–48. doi: 10.1006/nlme.1999.3919

6. Gaberel T, Gakuba C, Hebert M, Montagne A, Agin V, Rubio M, et al. (2013) Intracerebral hematomas disappear on T2*-weighted images during normobaric oxygen therapy. Stroke 44:3482–3489. doi: 10.1161/STROKEAHA.113.002045

7. Hébert M, Anfray A, Chevilley A, Martinez de Lizarrondo S, Quenault A, Louessard M, et al. (2017) Distant Space Processing is Controlled by tPA-dependent NMDA Receptor Signaling in the Entorhinal Cortex. Cereb Cortex N Y N 1991 27:4783–4796. doi: 10.1093/cercor/bhw275

8. Hébert M, Bulla J, Vivien D, Agin V (2017) Are Distal and Proximal Visual Cues Equally Important during Spatial Learning in Mice? A Pilot Study of Overshadowing in the Spatial Domain. Front Behav Neurosci 11:109. doi: 10.3389/fnbeh.2017.00109

9. Isingrini E, Camus V, Le Guisquet A-M, Pingaud M, Devers S, Belzung C (2010) Association between repeated unpredictable chronic mild stress (UCMS) procedures with a high fat diet: a model of fluoxetine resistance in mice. PLoS One 5:e10404. doi: 10.1371/journal.pone.0010404

10. Isingrini E, Surget A, Belzung C, Freslon J-L, Frisbee J, O’Donnell J, et al. (2011) Altered aortic vascular reactivity in the unpredictable chronic mild stress model of depression in mice: UCMS causes relaxation impairment to ACh. Physiol Behav 103:540–546. doi: 10.1016/j.physbeh.2011.04.002

11. Mineur YS, Belzung C, Crusio WE (2006) Effects of unpredictable chronic mild stress on anxiety and depression-like behavior in mice. Behav Brain Res 175:43–50. doi: 10.1016/j.bbr.2006.07.029

12. Mizoguchi K, Yuzurihara M, Ishige A, Sasaki H, Tabira T (2002) Chronic stress impairs rotarod performance in rats: implications for depressive state. Pharmacol Biochem Behav 71:79–84. doi: 10.1016/S0091-3057(01)00636-0

13. Nollet M, Le Guisquet A-M, Belzung C (2013) Models of depression: unpredictable chronic mild stress in mice. Curr Protoc Pharmacol Chapter 5:Unit 5.65. doi: 10.1002/0471141755.ph0565s61

14. Obiang P, Macrez R, Jullienne A, Bertrand T, Lesept F, Ali C, et al. (2012) GluN2D Subunit-Containing NMDA Receptors Control Tissue Plasminogen Activator-Mediated Spatial Memory. J Neurosci 32:12726–12734. doi: 10.1523/JNEUROSCI.6202-11.2012

15. Obiang P, Maubert E, Bardou I, Nicole O, Launay S, Bezin L, et al. (2011) Enriched housing reverses age-associated impairment of cognitive functions and tPA-dependent maturation of BDNF. Neurobiol Learn Mem 96:121–129. doi: 10.1016/j.nlm.2011.03.004

16. Porsolt RD, Bertin A, Jalfre M (1977) Behavioral despair in mice: a primary screening test for antidepressants. Arch Int Pharmacodyn Ther 229:327–336

17. Reynolds CD, Jefferson TS, Volquardsen M, Pandian A, Smith GD, Holley AJ, et al. (2017) Oral aniracetam treatment in C57BL/6J mice without pre-existing cognitive dysfunction reveals no changes in learning, memory, anxiety or stereotypy. F1000Research 6:1452. doi: 10.12688/f1000research.11023.3

18. Robison LS, Popescu DL, Anderson ME, Beigelman SI, Fitzgerald SM, Kuzmina AE, et al. (2018) The effects of volume versus intensity of long-term voluntary exercise on physiology and behavior in C57/Bl6 mice. Physiol Behav 194:218–232. doi: 10.1016/j.physbeh.2018.06.002

19. Strekalova T, Spanagel R, Bartsch D, Henn FA, Gass P (2004) Stress-induced anhedonia in mice is associated with deficits in forced swimming and exploration. Neuropsychopharmacol Off Publ Am Coll Neuropsychopharmacol 29:2007–2017. doi: 10.1038/sj.npp.1300532

20. Vollmer LE, Ghosal S, A Rush J, R Sallee F, P Herman J, Weinert M, et al. (2013) Attenuated stress-evoked anxiety, increased sucrose preference and delayed spatial learning in glucocorticoid-induced receptor-deficient mice. Genes Brain Behav 12:241–249. doi: 10.1111/j.1601-183X.2012.00867.x
