# Supplementary figures and images for: Plasminogen Activator Inhibitor-1 (PAI-1) deficiency predisposes to depression and resistance to treatments
Source: Acta Neuropathol Commun. 2019 Oct 14;7:153. doi: 10.1186/s40478-019-0807-2 (PMC6791031; doi:10.1186/s40478-019-0807-2)

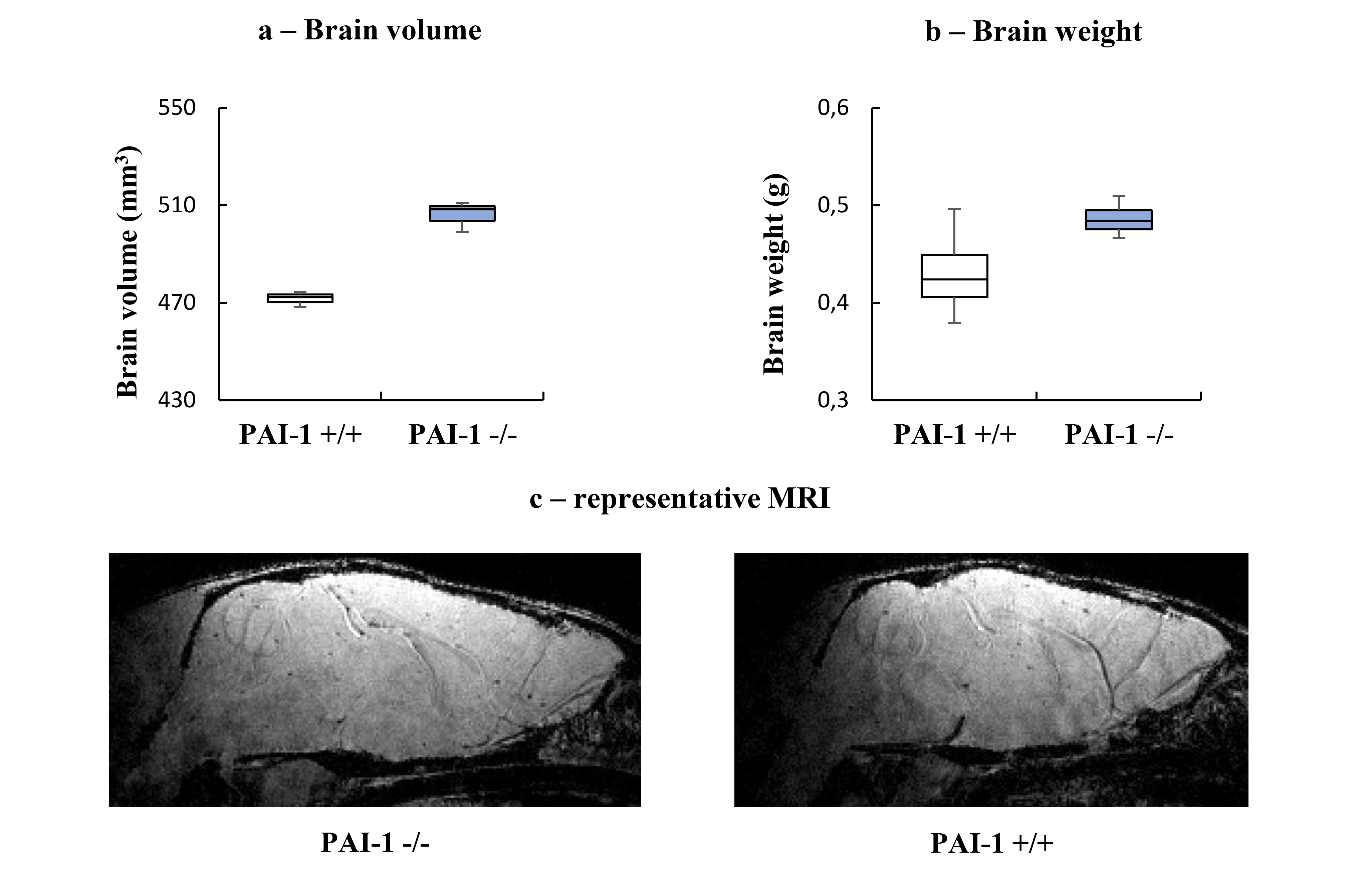

Supplement: Supplementary file 2 — Additional file 2: Figure S1. The depressive-like phenotype of PAI-1 −/− mice is not attributed to any brain weight or volume disparity. Quantitative analyses of brain volume (a) and weight (b) in PAI-1 knockout mice (PAI-1 −/−) and of their wild-type littermates (PAI-1 +/+). (c) Representative T2-weighted images of PAI-1 −/− and PAI-1 +/+ mice’s brain. n = 3 for each group. Mann-Whitney U-tests: P > 0.05. Boxplots show distributions with black horizontal lines indicating the median, box margins denoting the lower and upper quartiles. Whiskers show the minimum and maximum values. [file 40478_2019_807_MOESM2_ESM.tif]

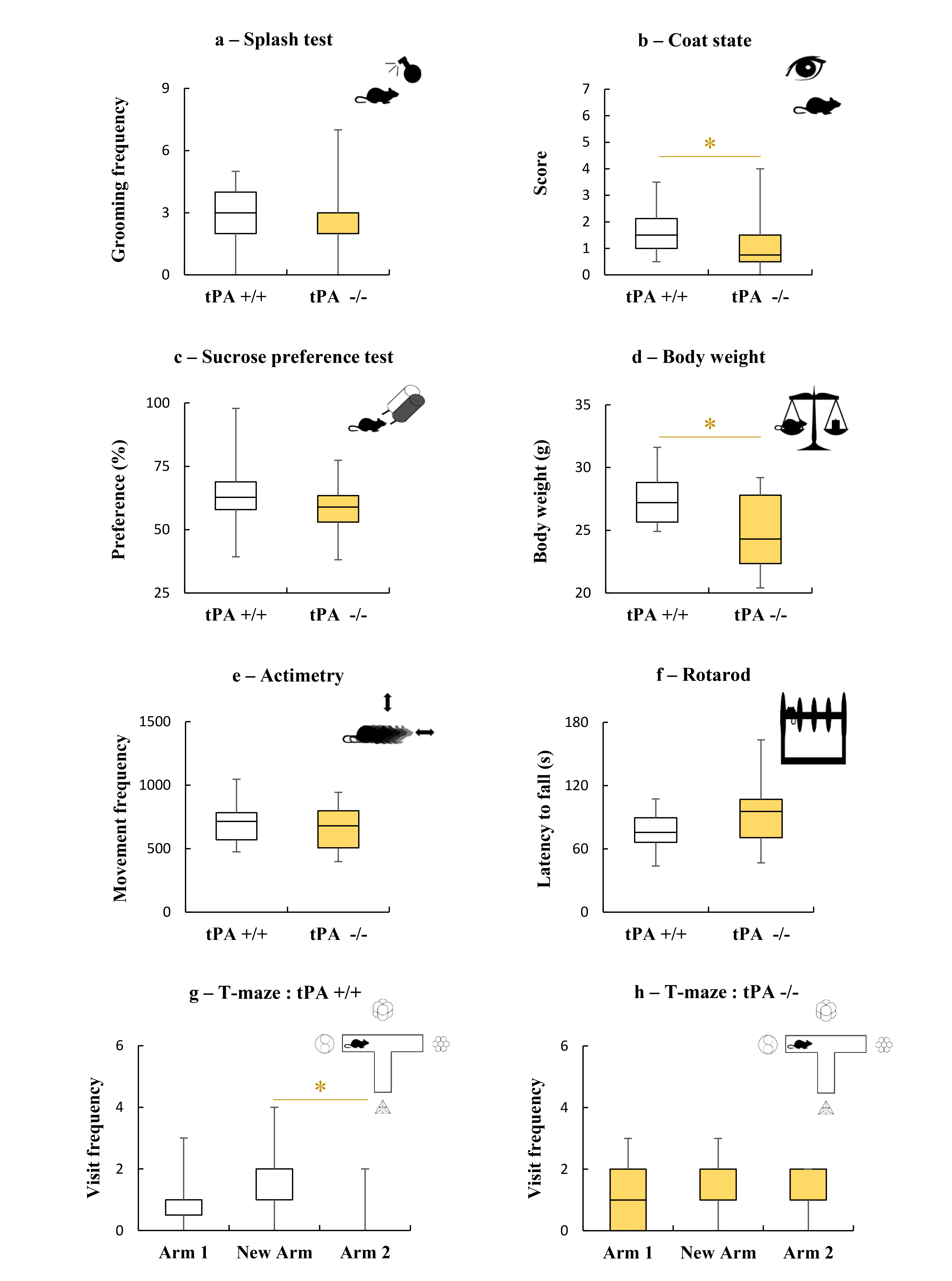

Supplement: Supplementary file 3 — Additional file 3: Figure S2. tPA deficiency does not induce a depressive-like phenotype. Evaluation of the behavioral phenotype of tPA knockout mice (tPA−/−) and of their wild-type littermates (tPA+/+). (a) Splash test: ntPA+/+ = 20; ntPA−/− = 20. (b) Coat state: ntPA+/+ = 20; ntPA−/− = 20. (c) Sucrose preference test: ntPA+/+ EE = 20; ntPA−/− EE = 20. (d) Body weight: ntPA+/+ EE = 15; ntPA−/− = 15. (e) Actimetry: ntPA+/+ = 19; ntPA−/− = 20. (f) Rotarod: ntPA+/+ = 20; ntPA−/− = 20. (g-h) T-maze: ntPA+/+ = 15; ntPA−/− = 13. Mann-Whitney U-tests (a-b), Student t tests (c-f), Wilcoxon signed-rank tests (g-h): *P < 0.05. Boxplots show distributions with black horizontal lines indicating the median, box margins denoting the lower and upper quartiles. Whiskers show the minimum and maximum values. [file 40478_2019_807_MOESM3_ESM.tif]

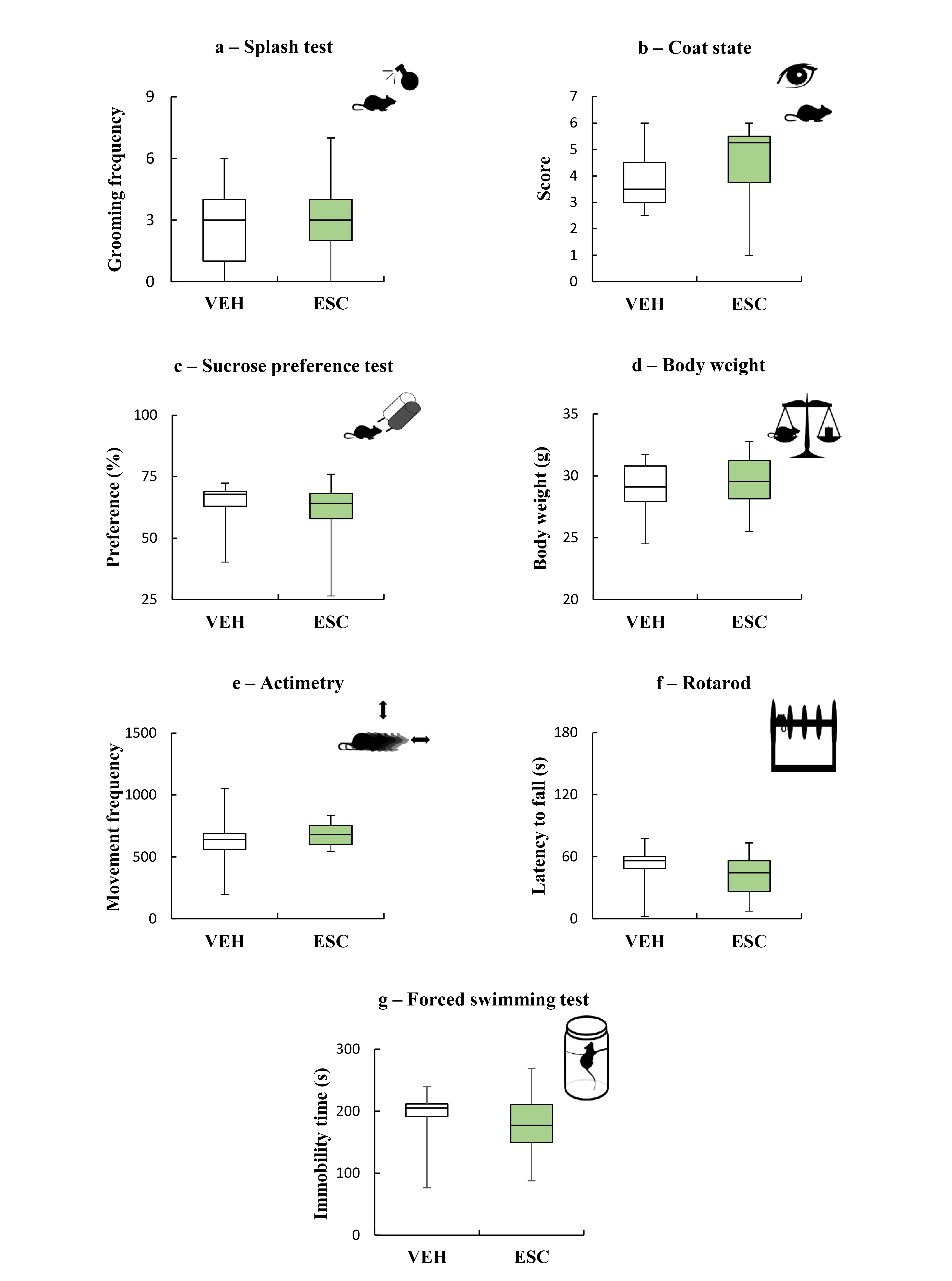

Supplement: Supplementary file 4 — Additional file 4: Figure S3. PAI-1 knockout mice fail to respond to escitalopram treatment. Evaluation of the behavioral phenotype of PAI-1 knockout mice (PAI-1−/−) submitted to an escitalopram chronic treatment (35 days) at 30 mg/kg. Escitalopram: ESC; Vehicle (NaCl 0.9%): VEH. (a) Splash test: nVEH = 12; nESC = 12. (b) Coat state: nVEH = 12; nESC = 12. (c) Sucrose preference test: nVEH = 12; nESC = 12. (d) Body weight: nVEH = 12; nESC = 12. (e) Actimetry: nVEH = 12; nESC = 12. (f) Rotarod: nVEH = 12; nESC = 12. (g) Forced swimming test: nVEH = 12; nESC = 11. Mann-Whitney U-tests (a, c, e), Student t tests (b, d, f, g): P > 0.05. Boxplots show distributions with black horizontal lines indicating the median, box margins denoting the lower and upper quartiles. Whiskers show the minimum and maximum values. [file 40478_2019_807_MOESM4_ESM.tif]

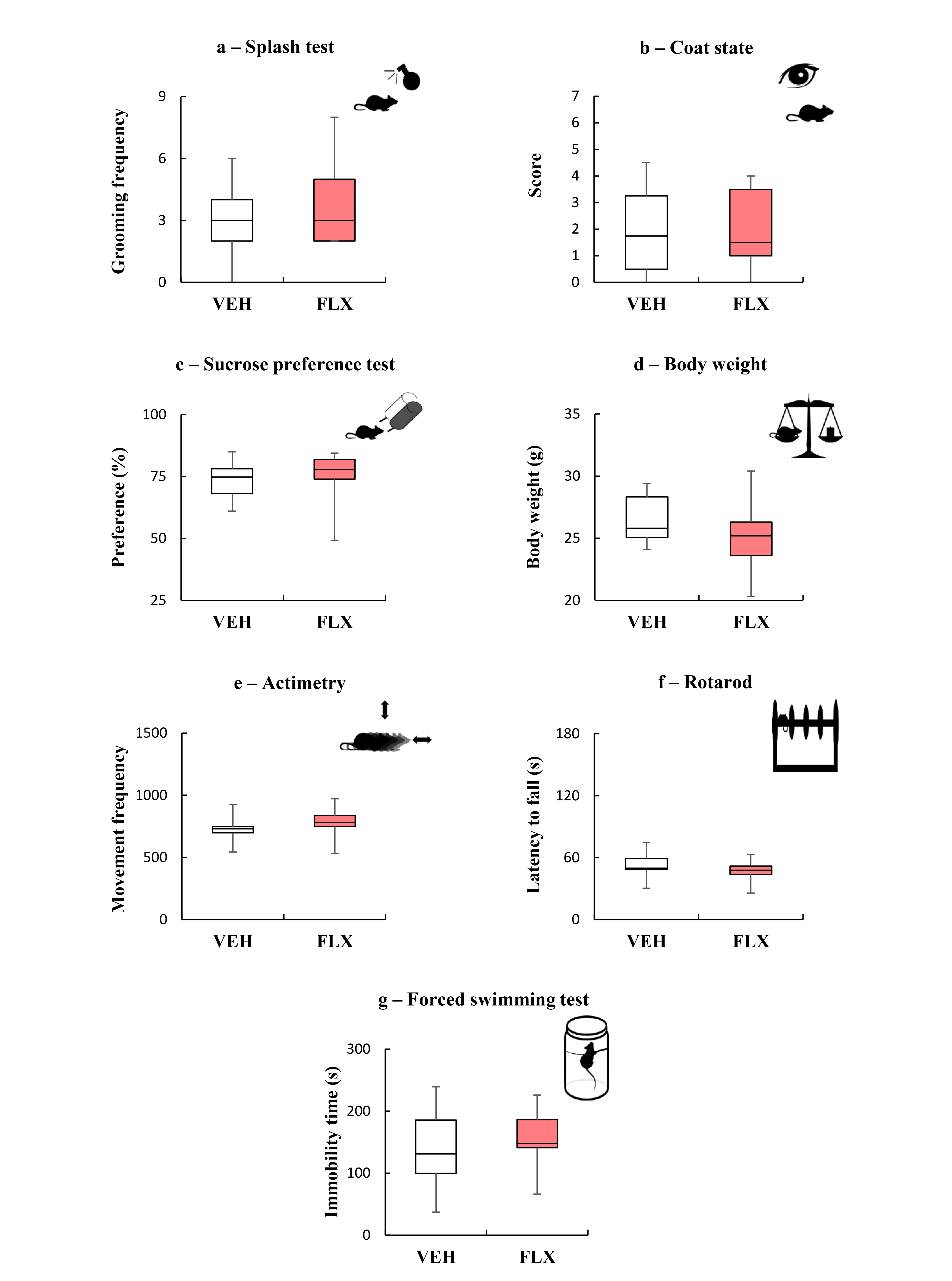

Supplement: Supplementary file 5 — Additional file 5: Figure S4. PAI-1 knockout mice fail to respond to fluoxetine treatment. Evaluation of the behavioral phenotype of PAI-1 knockout mice (PAI-1−/−) submitted to a fluoxetine chronic treatment (35 days) at 15 mg/kg. Fluoxetine: FLX; Vehicle (NaCl 0.9%): VEH. (a) Splash test: nVEH = 12; nFLX = 13. (b) Coat state: nVEH = 12; nFLX = 13. (c) Sucrose preference test: nVEH = 11; nFLX = 13. (d) Body weight: nVEH = 12; nFLX = 13. (e) Actimetry: nVEH = 12; nFLX = 13. (f) Rotarod: nVEH = 10; nFLX = 11. (g) Forced swimming test: nVEH = 12; nFLX = 12. Mann-Whitney U-tests (a-c, g), Student t tests (d-f): P > 0.05. Boxplots show distributions with black horizontal lines indicating the median, box margins denoting the lower and upper quartiles. Whiskers show the minimum and maximum values. [file 40478_2019_807_MOESM5_ESM.tif]
