# Supplementary material for: Plasminogen Activator Inhibitor-1 (PAI-1) deficiency predisposes to depression and resistance to treatments
Source: Acta Neuropathol Commun. 2019 Oct 14;7:153. doi: 10.1186/s40478-019-0807-2 (PMC6791031; doi:10.1186/s40478-019-0807-2)
Supplement: Supplementary file 6 — Additional file 6: Table S1. Number of mice used in the study. [file 40478_2019_807_MOESM6_ESM.docx]

**Table S1:** Strain, number and age of mice used in the study.

| **Behavioral experiments** | | | | |
| --- | --- | --- | --- | --- |
| **Behavioral tests / Genotype** | ***PAI-1 +/+ mice***  ***(10 weeks)*** | ***PAI-1 -/- mice***  ***(10 weeks)*** | ***tPA +/+ mice***  ***(10 weeks)*** | ***tPA -/- mice***  ***(10 weeks)*** |
| ***Splash test*** | n = 34 | n = 41 | n = 20 | n = 20 |
| ***Sucrose preference test*** | n = 35 | n = 39 | n = 20 | n = 20 |
| ***Body weight*** | n = 15 | n = 15 | n = 15 | n = 15 |
| ***Actimetry*** | n = 35 | n = 42 | n = 19 | n = 20 |
| ***Rotarod*** | n = 33 | n = 40 | n = 20 | n = 20 |
| ***Coat test*** | n = 35 | n = 42 | n = 20 | n = 20 |
| ***T-maze*** | n = 12 | n = 9 | n = 15 | n = 13 |

| **Molecular experiments** | | |
| --- | --- | --- |
| **Methods / Genotype** | ***PAI-1+/+ mice***  ***(10 weeks)*** | ***PAI-1-/- mice***  ***(10 weeks)*** |
| **UHPLC-MS/MS** | n = 5 | n = 5 |
| **Fibrin-agar zymography assay** | n = 5 | n = 5 |
| **Immunoblotting** | n = 5 | n = 5 |

| **Pharmacological experiments: Escitalopram (15 mg / kg)** | | | | |
| --- | --- | --- | --- | --- |
| **Behavioral tests / Genotype** | ***PAI-1+/+ mice + VEH***  ***(10 weeks)*** | ***PAI-1+/+ mice + ESC***  ***(10 weeks)*** | ***PAI-1-/- mice + VEH***  ***(10 weeks)*** | ***PAI-1-/- mice + ESC***  ***(10 weeks)*** |
| ***Splash test*** | n = 13 | n = 13 | n = 17 | n = 18 |
| ***Sucrose preference test*** | n = 13 | n = 13 | n = 17 | n = 18 |
| ***Body weight*** | n = 12 | n = 13 | n = 17 | n = 18 |
| ***Actimetry*** | n = 11 | n = 13 | n = 17 | n = 17 |
| ***Rotarod*** | n = 12 | n = 11 | n = 11 | n = 12 |
| ***Coat test*** | n = 13 | n = 13 | n = 17 | n = 18 |
| ***Forced swimming test*** | n = 13 | n = 13 | n = 17 | n = 17 |
| **Pharmacological experiments: Escitalopram (30 mg / kg)** | | | | |
| **Behavioral tests / Genotype** | ***PAI-1+/+ mice + VEH***  ***(10 weeks)*** | ***PAI-1+/+ mice + ESC***  ***(10 weeks)*** | ***PAI-1-/- mice + VEH***  ***(10 weeks)*** | ***PAI-1-/- mice + ESC***  ***(10 weeks)*** |
| ***Splash test*** | n = 10 | n = 10 | n = 12 | n = 12 |
| ***Sucrose preference test*** | n = 11 | n = 10 | n = 12 | n = 12 |
| ***Body weight*** | n = 11 | n = 12 | n = 12 | n = 12 |
| ***Actimetry*** | n = 11 | n = 11 | n = 12 | n = 12 |
| ***Rotarod*** | n = 11 | n = 10 | n = 12 | n = 12 |
| ***Coat test*** | n = 11 | n = 10 | n = 12 | n = 12 |
| ***Forced swimming test*** | n = 11 | n = 9 | n = 12 | n = 11 |

| **Pharmacological experiments: Fluoxetine (15 mg / kg)** | | | | |
| --- | --- | --- | --- | --- |
| **Behavioral tests / Genotype** | ***PAI-1+/+ mice + VEH***  ***(10 weeks)*** | ***PAI-1+/+ mice + FLX***  ***(10 weeks)*** | ***PAI-1-/- mice + VEH***  ***(10 weeks)*** | ***PAI-1-/- mice + FLX***  ***(10 weeks)*** |
| ***Splash test*** | n = 12 | n = 13 | n = 12 | n = 13 |
| ***Sucrose preference test*** | n = 11 | n = 12 | n = 11 | n = 13 |
| ***Body weight*** | n = 12 | n = 13 | n = 12 | n = 13 |
| ***Actimetry*** | n = 13 | n = 11 | n = 12 | n = 13 |
| ***Rotarod*** | n = 11 | n = 11 | n = 10 | n = 11 |
| ***Coat test*** | n = 12 | n = 13 | n = 12 | n = 13 |
| ***Forced swimming test*** | n = 13 | n = 11 | n = 12 | n = 12 |

UHPLC-MS/MS: Ultra-high-pressure liquid chromatography coupled with tandem-mass spectrometry; ESC: Escitalopram; VEH: Vehicle (NaCl 0.9%); FLX: Fluoxetine
