# Supplementary material for: Plasminogen Activator Inhibitor-1 (PAI-1) deficiency predisposes to depression and resistance to treatments
Source: Acta Neuropathol Commun. 2019 Oct 14;7:153. doi: 10.1186/s40478-019-0807-2 (PMC6791031; doi:10.1186/s40478-019-0807-2)
Supplement: Supplementary file 7 — Additional file 7: Table S2. Quantification modeling of depressive-like behaviors in mouse. [file 40478_2019_807_MOESM7_ESM.docx]

**Table S2:** Quantification modeling of depressive-like behaviors in mouse.

| **Depressive-like behaviors in mouse** | **Composite Score** |
| --- | --- |
| Apathetic behavior: Splash test | 1 |
| Anhedonic behavior: Sucrose preference test | 1 |
| Body weight | 1 |
| Hypoactivity or hyperactivity: Actimetry | 1 |
| Loss of motivation/effort: Rotarod | 1 |
| Deficits of self-centered behaviors: Coat state | 1 |
| Cognitive deficits: T-maze | 1 |
| ***Diagnosis of depressive-like phenotype:*** *At least 4 symptoms / 7 including apathetic and / or anhedonic behavior* | ***7*** |
