# Supplementary material for: Plasminogen Activator Inhibitor-1 (PAI-1) deficiency predisposes to depression and resistance to treatments
Source: Acta Neuropathol Commun. 2019 Oct 14;7:153. doi: 10.1186/s40478-019-0807-2 (PMC6791031; doi:10.1186/s40478-019-0807-2)
Supplement: Supplementary file 8 — Additional file 8: Table S3. Multiple Reaction Monitoring (MRM) method parameters. The MRM product ions in brackets are uses as confirmation transitions. [file 40478_2019_807_MOESM8_ESM.docx]

**Table S3:** Multiple Reaction Monitoring (MRM) method parameters. The MRM product ions in brackets are uses as confirmation transitions.

| ***Condition*** | ***5-HT*** | ***NA*** | ***DA*** | ***5-HMT*** | ***DHBA*** |
| --- | --- | --- | --- | --- | --- |
| ***RT (min)*** | 0.6 | 0.29 | 0.4 | 0.85 | 0.35 |
| ***MRM Precursor ion (m/z)*** | 177 | 170 | 154 | 191 | 140 |
| ***MRM Product ions (m/z)*** | 160.1 (115.2) | 152.0 (107.1) | 136.9 (118.9) | 160.1 (115.1) | 123.0 (105.1) |
| ***MRM CE (V)*** | -12 (-28) | -21 (-11) | -15 (-20) | -13 (-30) | -12 (-22) |

5-HT: Serotonin; NA: Noradrenaline; DA: Dopamine; 5-HMT: 5-hydroxy-N-*ω*-methyltryptamine; DHBA: 3,4-dihydroxybenzylamine; MRM CE : collision energy; RT : retention time
